# COVID-19 site readiness initiative: Building clinical trial capacity for vaccine efficacy trials in Latin America in response to the pandemic

**DOI:** 10.1016/j.jvacx.2022.100238

**Published:** 2022-11-08

**Authors:** Sue Ann Costa Clemens, Ana Keiko Sekine, Fernanda Tovar-Moll, Ralf Clemens

**Affiliations:** a Head of the Institute for Global Health, University of Siena, Italy; b Project Manager Consultant, Director Clinical Operations, Takeda Pharmaceuticals, Brazil; c Chief Executive Officer, Instituto D'Or de Pesquisa e Ensino, Brazil; d International Vaccine Institute IVI, Republic of Korea

**Keywords:** Capacity building, Clinical trial, Clinical trial site, COVID-19, Latin America, Vaccine trial

## Abstract

According to the World Health Organization, the American region has the highest coronavirus disease-2019 (COVID-19) cases and deaths since the start of the pandemic. This humanitarian tragedy presented the possibility of generating efficacy data from COVID-19 vaccine trials. The race to develop successful vaccines imposed a high demand for trained healthcare personnel and clinical sites where large scale randomized clinical trials could be conducted. This site readiness initiative, funded by the Bill and Melinda GatesFoundation (BMGF), was carried out to rapidly build site capacity for running COVID-19 vaccine trials in Latin America.

Twenty-two sites across 7 countries were selected and received funding. Site selection was based on defined feasibility criteria which deemed these sites as suitable for running vaccine efficacy trials. Criteria for selection included investigator and core permanent staff experience, public health measures in place for COVID-19, import/export requirements for study drug and biological specimens, a clear and accelerated ethical and regulatory approval process for COVID-19 trials. Training was tailored and delivered according to the experience level of the investigator and site staff, and included GCP training, standard operating procedures (SOP) fundamentals, conducting vaccine trials, COVID-19 pathophysiology, and vaccine trials lessons learned. Most of the grant funds were utilized for space expansion and renovation (46 %) followed by purchase of equipment (36 %); the remaining 18 % was spent on human resources. By the end of this site readiness initiative project, which took approximately 4 months, 21 of 22 (95 %) sites had agreements in place or were in discussions with sponsors to conduct large scale COVID-19 vaccine trials.

## Highlights

• BMGF launched a trial site readiness grant for COVID-19 clinical development.
• Latin America sites recently successfully executed various vaccine efficacy trials.
• Within 4 months 21 sites were ready to participate in COVID-19 vaccine studies.
• Infrastructure and equipment improvements consumed most of the sites’ budget (81%)
• This site readiness initiative may be a blueprint for public health emergencies.

## Introduction

Randomized clinical trials are considered the gold standard for assessing the safety and efficacy of new drugs and vaccines. Historically, high- and to a lesser extent high-middle income countries (HICs and HMICs) have been the biggest contributors of clinical trial data, but over the past decade many lower middle-income countries (LMICs) have received significant investments in a bid to develop capacity and infrastructure to conduct vaccine trials. Even so, some HMICs and LMICs still struggle to build sustainable capabilities or provide qualified regulatory oversight for conducting vaccine trials and maintain adequate infrastructure, thereby often needing the support of product development partners (PDPs) [1], [2].

In December 2019, a large number of people in Wuhan were reported to have pneumonia of an unknown origin which was subsequently identified as an epidemic due to a new strain of virus called severe acute respiratory syndrome coronavirus-2 (SARS-CoV-2). On 11th March 2020, the World Health Organization (WHO) announced this outbreak as a pandemic (coronavirus disease-2019 [COVID-19]), having spread across 6 continents [3], [4], [5]. The situation demanded immediate evidence-based research, calling for global collaborative efforts in the preparation and conduct of randomized controlled vaccine trials to provide evidence for controlling the pandemic [6].

As of 2nd December 2021, the current number of COVID-19 trials registered at ClinicalTrials.gov stands at 3,939. According to the WHO [7], [8], there were 140 candidate vaccines for COVID-19 under clinical development.

The global disease burden COVID-19 stands at 628 million cases and over 18 million deaths [9]. Despite the recent declining trend, the American region has the largest disease burden (37 % of total cases and 44 % of total deaths) with the highest number of new cases and deaths reported from the United States of America (USA), Brazil and Mexico [10].

Latin America has a tradition of vaccine development, having participated in recent years in large efficacy trials including the Rotavirus vaccines, the human papillomavirus vaccines (HPV), the Pneumococcal Conjugate vaccine as well as the two recent Dengue vaccine trials [11], [12], [13], [14], [15]. The region offers attractive development conditions for vaccine clinical trials owing to well-trained physicians and staff especially in infectious diseases, the high acceptance rate of vaccines, the distinct environment and ethnic diversity [16], [17], population density greater than the USA or Europe [18], dense urban areas [19], high-quality standards for conducting clinical trials, strict regulatory guidelines based on Good Clinical Practice (GCP) principles, and people, physicians and researchers willing to participate in clinical trials [20]. However, each country within Latin America has its own regulations (some with significant bureaucracy) and all lack research funds, thus making it challenging to maintain the infrastructure and capacity to conduct clinical trials [21], [22]. Unfortunately, without a constant flow of studies, following successful efficacy vaccine trials in the more distant and recent past, many sites were dismantled and lost qualified personnel, development capacity and site qualification [21].

With the COVID-19 pandemic spreading, and Latin America, in particular Peru, Brazil and Mexico, becoming hotspots, there was a rapid surge in demand for large scale COVID-19 vaccine trials for which the region and the world were not fully prepared. When the COVID-19 pandemic was declared (on 11 March 2020), nearly half (∼44 %) of the 7770 vaccine clinical trials registered on ClinicalTrials.gov were conducted in North America with only 6 % conducted in Latin America. By 31 July 2020, just before this initiative started, 131 COVID-19 vaccine trials were registered on ClinicalTrials.gov globally, with 20 (15 %) being conducted in Latin America [23].

Public health measures to contain the pandemic had an impact on site infrastructure, space needed to ensure social distancing and personal protective equipment demands. Staffing levels had to be rapidly expanded and personnel trained in the conduct of large vaccine trials for which recruitment in record time was crucial. Lockdowns and a high demand for health care professionals to ensure medical care aggravated the accessibility of qualified site personnel, a key factor for ensuring quality in clinical trials. Training on GCP, local regulations, clinical study protocols, and SOPs was essential as well as direct and frequent communication with the sites. Furthermore, specific equipment demands varied between trials and vaccine type, so ensuring continuous access to supplies during this global shortage crisis posed an added challenge.

Some of the key factors that define exemplary trial sites include fast recruitment, maintenance of high GCP standards and quality, and multidisciplinary involvement in the process [24], [25]. In addition to general clinical trial requirements, COVID-19 vaccine trials have specific needs such as infrastructure and additional personnel to cope with a high and fast recruitment never experienced by any site before. The expectation was to recruit 800-–1000 subjects per site per month. Furthermore, most sites were more experienced in paediatric trials than in adult trials, but adults were the initial target group for the development of the COVID-19 vaccines. This high enrollment impacts on many aspects: the need for a high number of site personnel proficient in remote data capture, including electronic diary cards to capture events in real time; specific storage and preparation requirements as some vaccines needed reconstitution and some mRNA vaccines needed transportation and storage at −70 °C thus demanding very rigorous cold chain control and specific freezers; logistics and personnel for processing large sample volumes; clinical trials material and sample shipment logistics in times when many flight routes were cancelled; and reduction in lead time for defining the site, training staff; recruiting and vaccinating participants, and in particular capabilities and resources for a diligent safety follow up as the most advanced vaccines were based on novel technologies, such as mRNA or viral vectors, with a very limited safety record [26].

Conscious that operational readiness focusing on the conduct of efficacy trials was paramount for a quick and successful development of COVID-19 candidate vaccines, BMGF established the COVID-19 site readiness initiative to help trial sites in Latin America, Africa, and Asia prepare and enhance their capabilities for conducting large scale COVID-19 vaccine trials with a high enrolment strategy and as per local and international guidelines. The initiative funded 3 PDPs: Instituto D’OR de Pesquisa e Ensino (IDOR), Rio de Janeiro, Brazil; the Program for Appropriate Technology in Health (PATH), Seattle, USA; and the International Vaccine Institute (IVI), Seoul, Korea. PATH and IVI covered the site readiness in Africa and Asia. This paper discusses how quick and efficient this Latin America COVID-19 site readiness initiative, the principal investigator of the grant and lead author, and IDOR, Brazil, as the PDP, was at building site capacity for COVID-19 vaccine trials in this region.

## Methods

A grant proposal (ID: INV-021464, total amount US$1,610,000) was approved by BMGF [27] to provide funds to IDOR, one of the PDPs, for preparing clinical trial sites to run large scale COVID-19 vaccine trials in Latin America. The implementation of this site readiness initiative was planned to last approximately 4 months (August 2020 to November 2020). The funding was intended for preparing or expanding trial sites in terms of infrastructure, equipment, staff, and training, for participating in COVID-19 vaccine trials, helping sites to expedite and ensuring quality of the vaccine trials.

### Selection of sites

The purpose of this initiative was to build/ improve and qualify clinical trial sites for conducting large scale Phase 3 COVID-19 vaccine trials mainly in adults within as early as 4 weeks after approvals for the most experienced sites.

To select sites for building capacity to conduct large scale vaccine trials, a feasibility form was developed for site selection and sent out to various sites in Latin America. These sites were identified through a combination of avenues: PDṔs previous clinical trials experience with sites; through VacciNet [28], a network of investigators and clinical trial sites in Latin America; screening registries for vaccine publications; knowledge of trials already conducted in the region; and via contact with clinical research organizations (CROs).

**Fig. 1:**
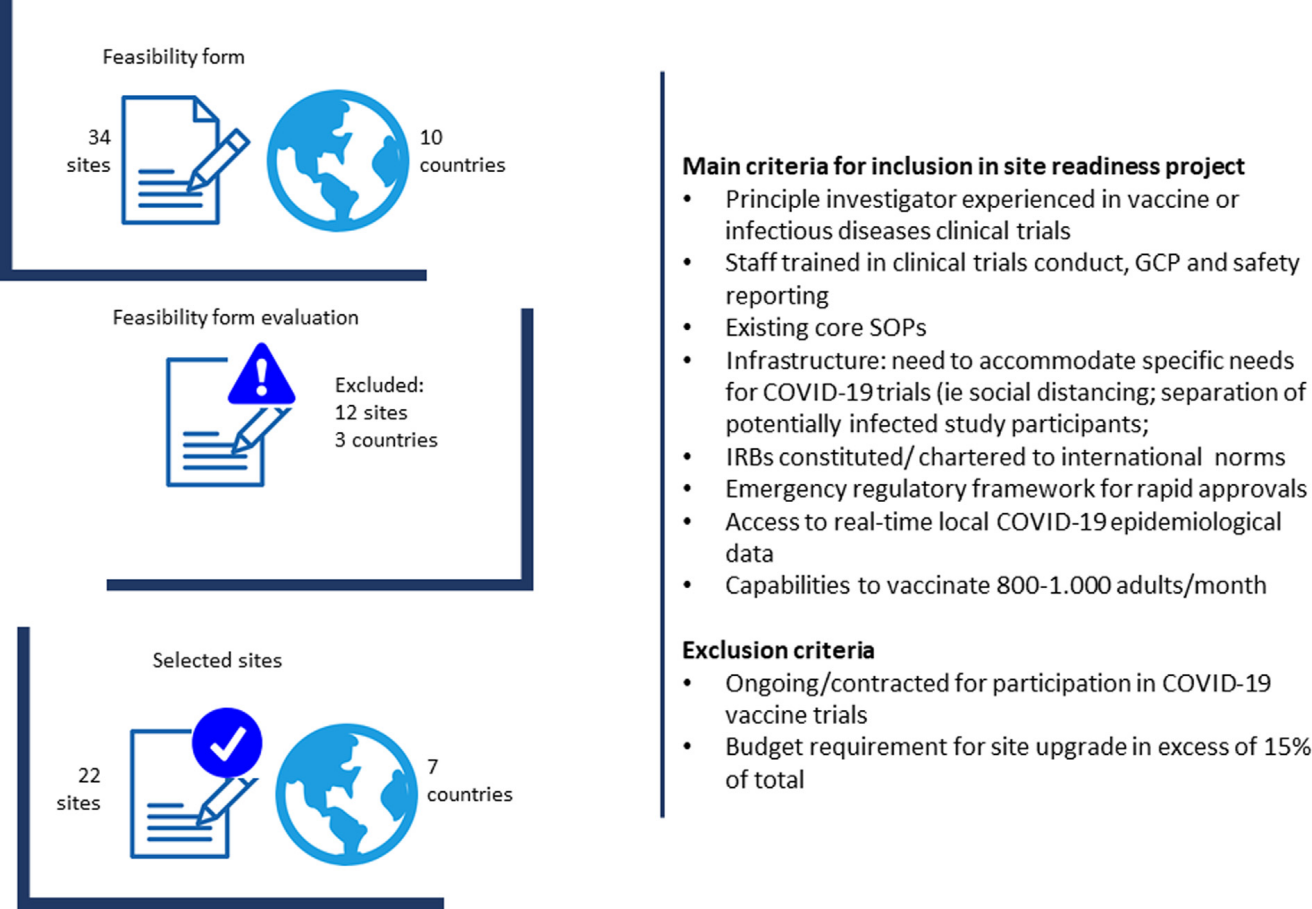
**Site selection flow chart.** Abbreviations: COVID-19 coronavirus disease-2019; GCP Good Clinical Practice; SOP standard operating procedures.

**Fig. 2:**
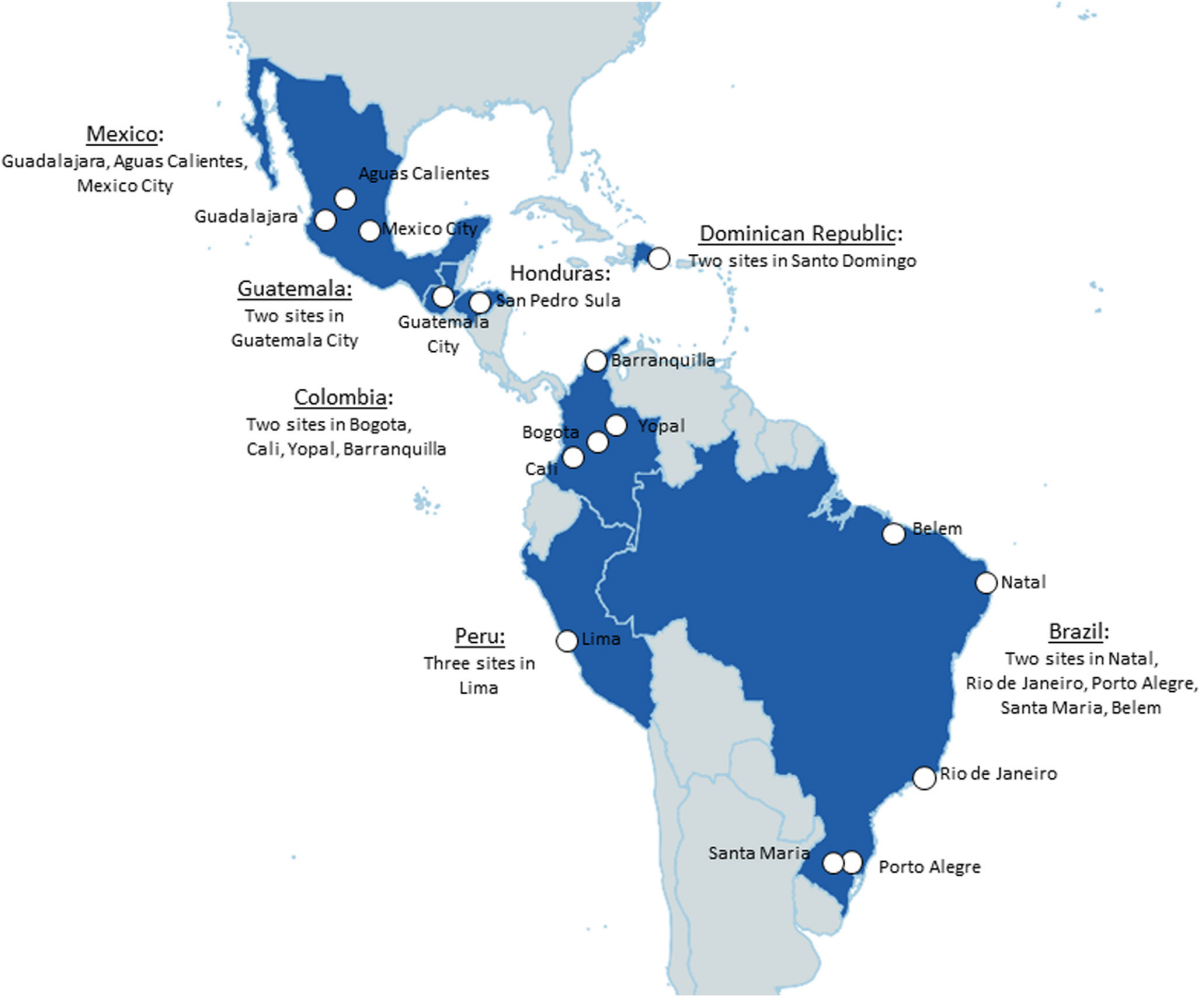
Map of selected sites. Modified from chart generated at https://www.mapchart.net/, using the Winkel Tripel map projection.

Main criteria for the selected sites to receive funding included experience of site and principle investigator in conducting clinical trials with focus on vaccine or infectious diseases, well trained staff including in GCP and safety reporting; existing SOPs; existing or potential to rapidly improve the infrastructure to accommodate the specific needs for COVID-19 trials, such as social distancing and separate space for work-up of potentially infected study participants; ethical review by Institutional Review Boards (IRBs) constituted and chartered to standard norms, regulatory approval timelines and logistics to allow a quick study start; capabilities to vaccinate 800–1.000 adult participants within a month, and access to real-time and reliable COVID-19 epidemiological data for their capture areas (selection criteria and flow chart in Fig. 1, Fig. 2).

The principal investigator of the grant and the management team reviewed the completed feasibility forms, considering and weighting various factors for final site selection. Experience in vaccine clinical trial conduct, internationally accepted ethical and regulatory standards and oversight, as well as time required to be ready and include the first subject in a COVID-19 vaccine trial were the key selection factor above the other deliverables, such as infrastructure, personnel involved, budget requirements, epidemiological data, processes followed, and site retention strategy.

The final site list was shared and agreed with the project funder, BMGF.

### Implementation phase

Due diligence was done for all selected sites to map current site capacity and experience, identify gaps and resource needs, check their budget requirements, and negotiate budget funding, after which contractual agreements were executed, and funding implemented. Funding included the purchase of site and laboratory equipment (such as fridges, freezers, and centrifuges) and administrative materials (such as desktops, laptops, broadband connection and Wi-Fi routers, secure servers and connection), the construction or renovation of physical space, the hiring of staff, the delivery of training sessions, the monitoring of epidemiological data, and the external site validation, and site security.

To assess training needs, the selected sites were classified in 3 tiers based on staff experience in clinical and vaccine trials, site facilities and equipment, priority and speed to initiate COVID-19 vaccine trials. Depending on this classification, tailored training sessions were prepared and delivered to all sites in preparation for the conduct of COVID-19 vaccine trials.

Training sessions varied in scope, subject depth, and length according to each tier’s needs. The content and training were developed and approved under the supervision of the University of Siena, Italy, which pioneered the first internationally recognized Master in Vaccinology and Pharmaceutical Clinical Development (https://ifgh.org/educational-programs/masters/master-invaccinology/) and has specific modules and materials for this training which include key subjects such as GCP, disease awareness, COVID-19 vaccine clinical trials fundamentals, vaccine clinical trials fundamentals recruitment strategies and adherence, data management – CRF and monitoring, finance, investigational product (IP) and laboratory management, safety reporting, shipment, regulatory reports, inspections, and SOP for basic studies. Trained site personnel received a certificate from the University of Siena.

To continuously assess the appropriateness of selected sites while the pandemic evolved, the following activities were additionally performed: (i) collection of COVID-19 epidemiological data and follow-up on a continuous basis; (ii) identification and documentation of data sources by country; and (iii) periodic surveys of selected sites using the COVAX dashboard for sharing new information on the sites and COVID-19, with biweekly dashboard updates and posting on the public COVAX platform set up by the CEPI [29].

## Validation of selected sites

Validation activities started after completion of the implementation phase to ensure the quality of the execution, the site readiness and the appropriate use of funds.

Prior to validation visits the sites were to send in documentations including CV and training records of the principal investigator and site staff, documentation of the ethical and regulatory processes, site set up for safety follow up, a grant utilization report and a progress report documenting the activities carried out to address the initially identified gaps.

Validation was done face-to-face wherever possible and, if not, virtually for each site by qualified consulting agencies. The process included interviews with the principal investigator and key site staff as well as a physical or virtual facilities tour of each site for assessing the site’s infrastructure and equipment. Based on the document submission, interviews and facility tour, an assessment report was written for each site to document: if and how they used the grant to improve their capabilities, if any inaccuracies in grant utilization were identified, and the outcome of the assessment.

***Operational activities***

The management of the grant’s full budget and the general activities for the Latin American region were closely followed by the grant PI and her management team which was composed of one managing senior scientific director; one project manager; a CRO; an agency specialized in performing audits and inspections; and one operational manager.

The Operational manager ensured that all the data needed for the COVAX dashboard was gathered in a timely manner and was accurate; this included epidemiology, IT, reports, graphics, and metrics data. The finance and legal departments were responsible for setting up of contracts and grant wires with all selected sites.

The sites were classified into tiers and a tailored training agenda was developed per tier. Training agenda, content and materials were reviewed and approved by the grant PI and the University of Siena.

Throughout the project, regular updates were shared with BMGF and the other PDPs to ensure an open communication.

## Results

### Site selection

Overall, 34 sites were contacted across 10 countries for completion of the feasibility form. Of those, 22 (65 %) sites across 7 countries were included in the site readiness initiative: 3 sites in Mexico, 2 in Guatemala, 1 in Honduras, 2 in Dominican Republic, 5 in Colombia, 6 in Brazil, and 3 in Peru. The list of selected sites is provided in (Table 1**)**.

**Table 1:** Sites Selected for Grant Award.

**Country**	**City**	**Site Name**
1 Mexico	Guadalajara	CidVID Investigación Biomédica (iBiomed)
2 Mexico	Aguas Calientes	CidVID Investigación Biomédica (iBiomed)
3 Mexico	Mexico City	Centro de Atención y Investigación Medica
4 Guatemala	Guatemala City	Centro de Estudios Clínicos Salud Avanzada (main site)
5 Guatemala	Guatemala City	Hospital Roosevelt (satellite site)
6 Honduras	San Pedro Sula	Demedica
7 Dominican Republic	Santo Domingo	Fundacion Dominicana de Perinatología Pro-BEBE (main site)
8 Dominican Republic	Santo Domingo	Fundacion Dominicana de Perinatología Pro-BEBE (satellite site HMNSA)
9 Colombia	Bogota	Centro de Estudios en Infectología Pediátrica (CEIP)
10 Colombia	Cali	Centro de Estudios en Infectología Pediátrica (CEIP)
11 Colombia	Bogota	Centro de Atencion e Investigacion Medica (Caimed)
12 Colombia	Yopal	Centro de Atencion e Investigacion Medica (Caimed)
13 Colombia	Barranquilla	Clinica de la Costa
14 Brazil	Belem	Instituto Evandro Chagas
15 Brazil	Santa Maria	Universidade Federal de Santa Maria (UFSM)
16 Brazil	Porto Alegre	Hospital de Clinicas de Porto Alegre (HCPOA)
17 Brazil	Rio de Janeiro	Instituto D’OR de Pesquisa e Ensino (IDOR Gloria D’OR)
18 Brazil	Natal	Instituto Atena de Pesquisa Clinica
19 Brazil	Natal	Centro De Estudos E Pesquisas Em Moléstias Infecciosas
20 Peru	Lima	Instituto de Investigacion Nutricional
21 Peru	Lima	Investigaciones Médicas en Salud
22 Peru	Lima	Instituto de Medicina Tropical Alexander von Humboldt, Universidad Peruana Caetano Heredia

Twelve (35 %) sites from 3 countries were excluded as they did not fulfil the selection criteria (Fig. 1, Fig. 2).

***Characteristics of included sites based on feasibility questionnaire***

Critical to the selection of these sites was that all of those sites or their investigators had previous experience with vaccine trials or with clinical trials for infectious diseases within the previous 5 years. All had core permanent staff experienced in clinical trials and trained in GCP, the majority of the sites (83 %) also had access to temporary staff to cope with an increased demand in trials. Senior site personnel had, based on their level of experience, a level of autonomy in executing the trials, with the support of experienced coinvestigators and site coordinators.

Most of the selected sites also had had previous audits or inspections without any critical findings (89 %); All sites were supervised by independent IRBs prioritizing COVID-19 clinical trials and constituted according to international regulations; all sites had adequate processes in place and qualified staff to ensure safety follow up. The existing or upgraded infrastructure reflected the specific requirements of a COVID-19 trial. The regulatory authorities of the countries where the selected sites were based had special provision for expedited review of clinical trial applications. All sites had access to epidemiological data on the COVID-19 pandemic of the country and mostly also of the site region, and the countries where the sites are based had no restrictions on importing the investigational vaccine or exporting biological specimens. In all countries, experienced CROs were present. The selected sites had government support for conducting COVID-19 trials and had access to suitable participants to ensure the target recruitment of 800–1.000 participants within 1 month. Furthermore, most sites had server security (72 %) systems in place, available and tested shipment courier providers (94 %), and all had access to a network of laboratories.

***Funding and training of selected sites: Results from the implementation phase***

Following site selection, contracts were finalized and the agreed funds were provided. The classification in tiers was primarily bases on staff experience and training requirements: *8 sites in Tier 1 – Advanced (trained on disease, COVID-19 vaccine clinical trials and GCP), 6 sites in* Tier 2 – Intermediate (trained on disease, vaccine clinical trials, COVID-19 vaccine clinical trials, GCP, and SOP fundamentals), and 8 sites in Tier 3 – Basic (trained on disease, deeper knowledge on the fundamentals of vaccine clinical trials, safety, surveillance, data management and operational aspects, COVID-19 vaccine clinical trials, GCP, and SOP fundamentals).

Three of the 8 sites in Tier 1 had their training prioritized as they had previously been approached by sponsors to conduct COVID-19 vaccine trials and had to be ready within a shorter timeframe so as not to jeopardize their awards.

A total of 629 staff were trained and certified, including key site staff (site investigators, sub-investigators, coordinators, study operational managers, nurses, laboratory and data management personnel, safety, quality, surveillance and call center teams). Staff who already had a current valid GCP certificate did not need to take the GCP exam but had to participate in GCP workshops, SOP fundamentals, disease awareness and COVID-19 vaccine fundamentals in vaccine clinical trials and all other trainings. If participants did not pass the GCP exam, they had to repeat it to get the certification. For continuity, the training material was shared with investigators at all sites to ensure training of any staff who could not attend delivered sessions and to train any new staff recruited for upcoming trials. Qualified trainers were identified for this activity.

**Fig. 3:**
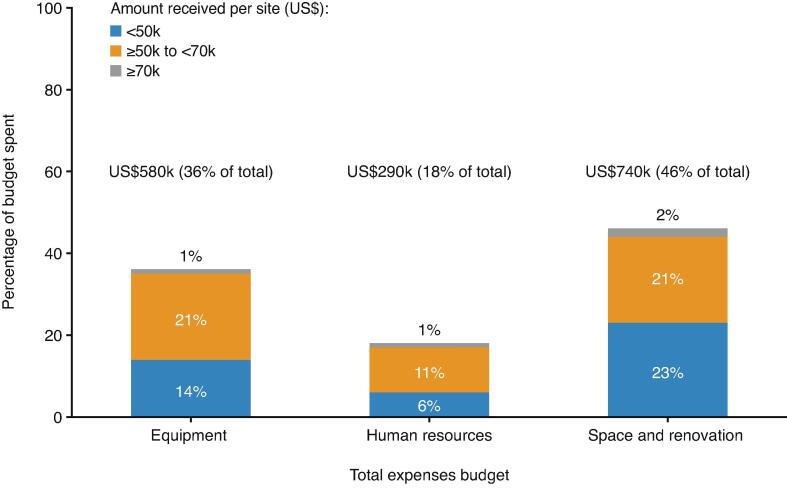
Grant Funds Utilization per Tier Allocation.

Grant funds were utilized for buying equipment, hiring human resources, and for building and renovating space, as per grant request agreement. See Fig. 3 for details on the usage of grant budget. Overall, the highest proportion of funds were used for building and renovating space (46 %) to meet the specific SARS-CoV-2 precautionary requirements and for buying equipment (36 %), and the remaining 18 % were used for human resources. The top categories reported were ‘medical consultation room’ under space and renovations; ultra-low temperature freezers (-80 °C), computers, power generators, freezers (-20 °C), and fridges (2 °C to 8 °C), under the equipment category; and biomedical/pharmacist/biologist, doctor, nurse, security, and site coordinator, under human resources. The individual grant dispensed to the sites ranged from US$35,000 to US$130,000.

## Performance of the selected sites: Results from the validation phase

A virtual tour was performed at all 22 sites and included checking of waiting rooms, consultation rooms, cold rooms, IP, management rooms, vaccination rooms, laboratories, machinery area (power and IT), offices and administrative spaces, designated archival area and satellite sites.

Key recommendations following from the virtual tours were: the creation of site SOPs for cold chain maintenance and contingency plans (8 [36 %] sites), clinical trial material transfer to satellite sites (3 [14 %] sites), and sample transfer to main site/laboratory (2 [9 %] sites). Other recommendations for site process for improvement were related to other SOP areas: informed consent form (ICF) process, site staff training, equipment maintenance, and calibration, contingency planning in case of a disaster, and clinical trial management in case of a pandemic. At the end of this site readiness initiative project, 21 of 22 (95 %) sites were ready to conduct COVID-19 vaccine trials as per the requirements of this project. Importantly, as the COVAX website was regularly updated with site readiness details, by the end of the project each of these 21 sites already had agreements in place or were in discussions with sponsors to conduct large scale COVID-19 vaccine trials.

The remaining site was a completely new site in Bogota, Colombia, that was created and is managed by CEIP, Investigational Center for Pediatric Infectious Diseases, based in Cali. The new CEIP site in Bogota was created solely for COVID trials using the budget received from this grant. This site was certified by the Colombian NRA in May 2021.

## Discussion

Since the COVID-19 pandemic was declared, an immense global collaborative effort has been made to advance science and stop, or at least control, its devastating effects on human health and health systems worldwide. The race to have an efficacious and safe vaccine available for a wider population, even if for emergency use only, was heightened. The high number of cases globally provided an important opportunity for efficacy trials to be conducted, but uncovered a lack of readiness to execute large studies in a short time in those areas which were most affected. This vital COVID-19 site readiness initiative, funded by BMGF, was set up to support and prepare clinical trials sites in HMICs and LMICs for the conduct of an unprecedented number of concurrent Phase 3 clinical trials with new COVID-19 specific infrastructure, physical capacity, and staffing requirements [29]. Considering its outcome, this initiative was highly successful. In total, 34 sites were mapped in 10 countries, and 22 (65 %) sites across 7 countries were selected to participate in this initiative. All 22 sites were ready to conduct large scale Phase 3 efficacy trials within 4 months of project start with one new site pending regulatory authority certification. This included 10 new independent investigational areas that were either the result of expansion of sites already located within the same building or independent satellite sites located in a different area from the main investigational site, created using this grant’s resources, and developed solely for the conduct of large vaccine trials. These totally new investigational sites/areas, now qualified for conducting large Phase 3 trials, are a legacy of the pandemic and are the result of a joint effort from BMGF, experienced research teams and local investigators that brings hope for development in time to help the pandemic.

**Fig. 4:**
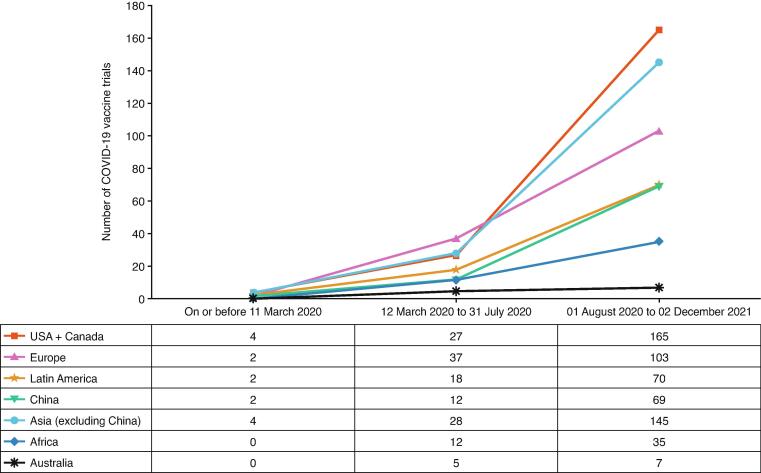
Rise in COVID-19 Vaccine Interventional Trials since the Beginning of the Pandemic.

**Fig. 5:**
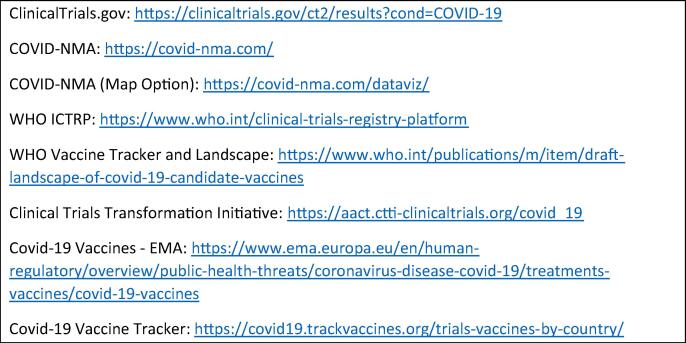
Websites on coronavirus disease-2019 (COVID-19) studies.

Despite the ongoing pandemic, all sites managed to get ready in both capacity building and infrastructure. Considering the number of COVID-19 vaccine trials ongoing in Latin America (Fig. 4) as of 2nd December 2021, we may assume that this initiative was a key contributor to the sharp rise in the numbers of such trials – a 4-fold increase from the time this initiative was kicked-off (Fig. 5 lists websites with information on COVID-19 studies).

So far, few studies have been conducted on strengthening of clinical trial capacity through infrastructure upgrades, recruitment, and personnel training, thereby leading to improved ability for conducting future trials and thus, better health systems [30], [31], [32], [33], [34], [35]. The present study, conducted in Latin America, is a first of its kind to focus on a rapid improvement in the capacity of clinical trial sites to run large scale vaccine trials, particularly considering the current global burden of the COVID-19 pandemic and the intrinsic challenges when dealing with respiratory infectious diseases. To successfully achieve the goals of this project in record time, we focused on: 1) appropriate site selection, guided and supported by the feasibility questionnaire created; 2) assessment of training needs and gaps, with development and delivery of training at the level required to each selected site; 3) securing and providing the grant budget requested by the sites for purchase of equipment and consumables. Of note, it was essential to have an experienced team in place to plan, implement and deliver the training, and meet the main objective of the project – to ensure timely vaccine development. The assessment of training needs and gaps, and delivery of tailored training to each site were critical to accomplish the high and fast subject enrolment required for Phase 3 COVID-19 vaccine trials. Recruitment conditions during a pandemic are much more strenuous and consuming on site staff, and training is key to guarantee not only recruitment but also quality and accuracy of data.

Conducting clinical trials at inadequate sites can lead to delays in starting trial related activities, under-recruitment, poor data quality. This can put study participants at risk, result in inefficiencies and wastage of time, cost, and resources, besides the high risk of invalidating the data collected and possibly even the whole trial and, in this case, delaying the hope for an authorized/licensed product to start controlling the pandemic. Thus, selecting a suitable clinical trial site and investigational team, getting it ready to run a clinical trial, and ensuring its performance are important steps to ensure the successful completion of a trial [36]. Further, investigator-dependent factors, such as previous experience, concurrent workload, and publications record, and ease of trial approval are critical factors that determine site selection [37]. Thus, a feasibility questionnaire was developed and sent to all Latin American mapped sites as a means of assessing their status, identifying gaps, and selecting sites that could create new areas or satellite sites, was crucial for expanding vaccine trials capacity in this region.

All sites and/or investigators were selected based on the robust feasibility characteristics such as experience in the conduct of vaccine trials or other clinical trials in infectious disease areas in the last 5 years, access to laboratories, public health measures for COVID-19 as per regulatory requirements, no restrictions to import trial drug or to export biological specimens, and access to risk group. GCP deviations are commonly found in the review process of new submissions, highlighting the need for in-depth and intensive training while preparing for clinical trials [38], [39]. Thus, the present initiative also had a special focus on GCP training for the site staff involved in the conduct of these trials. The site selection process was based on experienced CRO qualification processes of a site but had important differentiations: possibility for early and repetitive interaction with authorities with respect to study approvals, epidemiological data access, ease of import and export is not a standard procedure for a CRO. Timelines between selection, upgrade, qualification, and study start were very much compressed compared to standard CRO metrics. The specific requirements for a COVID-19 treatment or vaccine trial were to be respected and implemented. A virtual inspection of the sites was innovative based on the travel restrictions and social distancing requirements. The training of the site staff was tailor made for the experience of the site which is not standard practice. And finally the costs for the validation of 22 sites across 7 countries were materially less than typical CRO charges.

This is an original and unique initiative, in a time of no precedents in the history of clinical development, to prepare investigational sites to deliver support to bring vaccines to the population in record time, while the pandemic was ongoing. The site readiness project team, assembled in a short period of time by the lead author and grant PI, was crucial to achieve the goals of the project. This experienced team was balanced with different talents within clinical development to draw a very solid and thorough plan with strict timelines, implementation path, back-up solutions to respond to unexpected challenges and expected outcomes. The strategic plan was reflected in the deliverables and timelines - for each specific aspect, generating clusters of trainings and tailormade agendas to prepare and build sites with international standards for Phase 3 clinical development. All international and national guidelines with regards to GCP, ICH and local regulatory and ethical rules were respected. Thus, the newly created IDOR site readiness team for this project, together with the University of Siena, developed and tailored the training curricula according to the selected sites’ prior experience with vaccines/clinical trials, in line with the aim of preparing these sites for COVID-19 trials or vaccine trials in general. This also provided an academic supervision and certificates for those trained, thus enriching and improving their fundamental knowledge in this field of work. This type of specialized and tailor-made training is not generally universally available and therefore it was a key motivator for the sites and staff as well as for the local scientific communities and authorities in countries that supported this initiative either in-kind or actively.

A decision to validate the sites by an independent organization was taken to ensure that quality with regards to personnel and site infrastructure were met to cope with the international standards and that data generated in those sites would immediately contribute to regulatory dossiers for COVID-19 vaccine registrations. Those sites were inserted in the COVAX dashboard where the developers of the COVID-19 vaccines were able to search for qualified sites. The site validation was one more warranty for sponsors that the site selected would be immediately effective and produce quality and regulatory approvable data. Therefore, sponsors could focus on the product, submissions and clinical development plans to bring forward new products to the general population. As validation outcomes, minor aspects on SOP recommendations were highlighted, which ensured the quality training and readiness of both site and personnel. No major or critical aspects were highlighted during the validation. At the time of validation, the results confirmed that all sites had utilized the greatest amount of their grant (81 %) for site infrastructure improvement (equipment, and space and renovation), and the rest on human resources, to increase site enrolment capacity.

Part of the grant was reserved for hiring personnel once a study was due to start at the site or to pay the salaries of site personnel while negotiations with vaccine trial sponsors were still ongoing, to retain the trained personnel.

Several challenges were encountered during the conduct of this initiative, none of which caused a serious hindrance or delay to the project. Some of the countries initially mapped as potential participants had to be dropped due to lack of qualified personnel or because it would be too costly or time consuming to create the infrastructure required. Moreover, due to the ongoing pandemic and the need to accelerate vaccine development, selected sites were competing not only with healthcare systems but also with other sites, sponsors and CROs already running COVID-19 studies for qualified and experienced personnel. Attention had to be given to finding and retaining site personnel.

Considering the worldwide shortage of equipment and consumables, sites reported the same difficulties: freezers, centrifuges, laptops, PPE and other equipment that were either sold out or priced 4-10x the normal market value. Investigators and site personnel had to use their negotiating skills to get their goods in time and within granted budget; direct contact with distributors proved important to get them to commit to supplying the required goods.

The commitment, dedication, and enthusiasm of the investigators were crucial for success – even though they had no guarantee that they would be awarded the conduct of a COVID-19 vaccine trial.

Some of the limitations of this site readiness initiative were due to the restrictions imposed in this pandemic situation: the majority of the interactions with the sites were remote (online), particularly the feasibility assessments and validation activities; most sites had predominantly experience in paediatric vaccine trials and less so in adults; the urgency to have the sites ready to run clinical trials within a few weeks to months due to the high demand for COVID-19 trials; and the lack of a blueprint for this particular type of site readiness initiative in such a particular situation in which healthcare systems were already overloaded.

## Conclusion

To overcome the COVID-19 pandemic, fast vaccine development is crucial as a cornerstone in pandemic management. To develop a safe and efficacious vaccine, it must be tested through randomized controlled clinical trials with a large sample size. This site readiness initiative was carried out to build or improve clinical research sites to expand the capacity to conduct high-quality, large scale COVID-19 efficacy vaccine trials in Latin America, which had been a major contributor in these trials in the past. Through this initiative, suitable sites were selected in the Latin American region according to the feasibility criteria; gaps, mainly related to infrastructure, training and human resources, were identified, after which funding and training were implemented. Validation was performed to ensure those initially identified gaps were resolved. A total of 22 sites, including 10 new investigational sites, or areas within sites, were capacitated, validated and qualified within 4 months. The project was highly successful: 21 of the 22 (95 %) sites are currently involved in vaccine efficacy trials. In addition, this initiative also provided important information related to current barriers and their resolutions to enable the building of COVID-19 clinical trial sites in the Latin America region and worldwide. Hopefully, mechanisms will be put into place in order to keep those sites as reference of excellence in vaccine trials through continuous involvement in vaccine trials beyond COVID-19. One of the longer term effects of this grant project is further institutional strengthening which hopefully will be sustainable.

Clinical Trials are the primary way to generate actionable evidence for healthcare interventions. The COVID-19 response, led by initiatives such as BMGF site readiness, has demonstrated the critical importance of clinical trials, highlighting the need for continuous support for an international framework on capacity building in clinical development. International mechanisms for collaboration and coordination of clinical trials network must be strengthened. The sites that are part of this initiative need continuous investment to keep enhancing clinical trial capability, specifically in HMICs and LMICs, to ensure capacity with quality is available where it is most needed to better address ongoing global health issues and to respond rapidly to possible health threats.

This successful initiative will remain as one of the legacies of the COVID-19 pandemic. We hope that it can also serve as a blueprint on how to find, select, qualify and validate sites for clinical trials of different complexities in public health emergency situations.

**Funding sources**

This work was supported by the Bill and Melinda Gates Foundation, Seattle, Washington, USA (Grant No INV-021464).

**Data Sharing Statement**

The Bill and Melinda Gates Foundation group has regularly shared the project data via the COVAX website - Dashboard of COVAX-Supported Sites Ready for Clinical Trials (**https://epi.tghn.org/covax-overview/clinical-science/clinical/#ref**).

## Declaration of Competing Interest

The authors declare the following financial interests/personal relationships which may be considered as potential competing interests: [Sue Ann Costa Clemens is a Professor of Pediatrics Infectious Diseases, Head of the Institute for Global Health University of Siena and Director Vaccine Group Oxford-Brazil, Visiting Professor in Global Health at Oxford University, Department of Pediatrics and Senior Adviser and consultant at the Bill and Melinda Gates Foundation. Ana Keiko Sekine is a Director of Clinical Operations – COVID Vaccine Program, Moderna Inc. as of August 2021. Fernanda Tovar-Moll is the CEO and a member of the Board of Trustees of Instituto D’Or de Pesquisa e Ensino. Ralf Clemens is an adviser to BMGF and member of the Board of Trustees of IVI.].

## Data Availability

Data will be made available on request.

## References

[1] Dolgin E. (2010). African networks launch to boost clinical trial capacity. Nat Med.

[2] Grenham A., Villafana T. (2017). Vaccine development and trials in low and lower-middle income countries: Key issues, advances and future opportunities. Hum Vaccin Immunother.

[3] Gorbalenya A.E., Baker S.C., Baric R.S., De Groot R.J., Drosten C., Gulyaeva A.A. (2020).

[4] World Health O (2020).

[5] Chen N., Zhou M., Dong X., Qu J., Gong F., Han Y. (2020). Epidemiological and clinical characteristics of 99 cases of 2019 novel coronavirus pneumonia in Wuhan, China: a descriptive study. Lancet.

[6] Nasrallah A.A., Farran S.H., Nasrallah Z.A., Chahrour M.A., Salhab H.A., Fares M.Y. (2020). A large number of COVID-19 interventional clinical trials were registered soon after the pandemic onset: a descriptive analysis. J Clin Epidemiol.

[7] International Clinical Trials Registry Platform (ICTRP). https://www.who.int/clinical-trialsregistry-platform [Accessed on:2 Feb 2022].

[8] COVID-19 vaccine tracker and landscape. https://www.who.int/publications/m/item/draftlandscape-of-covid-19-candidate-vaccines [Accessed on:2 Feb 2022].

[9] World Health Organization. WHO Coronavirus (COVID-19) Dashboard. Available at: https://covid19.who.int/ Accessed on November 1st, 2022.

[10] WHO Coronavirus (COVID-19) Dashboard. https://covid19.who.int/ [Accessed on:2 Feb 2022].

[11] Velázquez R.F., Linhares A.C., Muñoz S., Seron P., Lorca P., DeAntonio R. (2017). Efficacy, safety and effectiveness of licensed rotavirus vaccines: a systematic review and meta-analysis for Latin America and the Caribbean. BMC Pediatr.

[12] Ruiz-Sternberg Á.M., Moreira E.D., Restrepo J.A., Lazcano-Ponce E., Cabello R., Silva A. (2018). Efficacy, immunogenicity, and safety of a 9-valent human papillomavirus vaccine in Latin American girls, boys, and young women. Papillomavirus Res.

[13] De Oliveira L.H., Camacho L.A.B., Coutinho E.S.F., Martinez-Silveira M.S., Carvalho A.F., RuizMatus C. (2016). Impact and Effectiveness of 10 and 13-Valent Pneumococcal Conjugate Vaccines on Hospitalization and Mortality in Children Aged Less than 5 Years in Latin American Countries: A Systematic Review. PLoS ONE.

[14] Villar L., Dayan G.H., Arredondo-García J.L., Rivera D.M., Cunha R., Deseda C. (2014). Efficacy of a tetravalent dengue vaccine in children in Latin America. N Engl J Med.

[15] Biswal S., Borja-Tabora C., Vargas L.M., Velásquez H., Alera M.T., Sierra V. (2020). Efficacy of a tetravalent dengue vaccine in healthy children aged 4–16 years: a randomised, placebo-controlled, phase 3 trial. Lancet.

[16] Salzano F.M., Sans M. (2014). Interethnic admixture and the evolution of Latin American populations. Genet Mol Biol.

[17] Norris E.T., Wang L., Conley A.B., Rishishwar L., Mariño-Ramírez L., Valderrama-Aguirre A. (2018). Genetic ancestry, admixture and health determinants in Latin America. BMC Genomics.

[18] World Population Prospects - Population Division - United Nations. https://population.un.org/wpp/ [Accessed on:2 Feb 2022].

[19] Urbanization-in-Latin-America-BBVA-Research. https://www.bbvaresearch.com/en/ [Accessed on:2 Feb 2022].

[20] L. Gómez H, A. Pinto J, Castañeda C, S. Vallejos C. Current barriers for developing clinical research in Latin America: A cross-sectional survey of medical oncologists. Clin Res Trials. 2015;1. https://doi.org/10.15761/crt.1000108.

[21] Alemayehu C., Mitchell G., Nikles J. (2018). Barriers for conducting clinical trials in developing countries- a systematic review. Int J Equity Health.

[22] Chomsky-Higgins K., Miclau T.A., Mackechnie M.C., Aguilar D., Avila J.R., dos Reis F.B. (2017). Barriers to Clinical Research in Latin America. Frontiers. Public Health.

[23] ClinicalTrials.gov. https://clinicaltrials.gov/ [Accessed on:2 Feb 2022].

[24] Baer A.R., Cohen G., Smith D.A., Zon R. (2010). Implementing clinical trials: a review of the attributes of exemplary clinical trial sites. J Oncol Pract.

[25] Zon R., Cohen G., Smith D.A., Baer A.R. (2011). Part 2: Implementing clinical trials: a review of the attributes of exemplary clinical trial sites. J Oncol Pract.

[26] Dean N.E., Gsell P.S., Brookmeyer R., De Gruttola V., Donnelly C.A., Halloran M.E. (2019). Design of vaccine efficacy trials during public health emergencies. Sci Transl Med.

[27] Instituto D'OR de Pesquisa e Ensino. https://www.gatesfoundation.org/about/committedgrants/2020/08/INV021464 [Accessed on:02 Feb 2022].

[28] VacciNet Vaccines Clinical Trials Network. https://vaccinet.org/index.php [Accessed on:2 Feb 2022].

[29] Epidemic Preparedness Innovations. COVAX. https://epi.tghn.org/covax-overview/clinicalscience/clinical/ [Accessed on:2 Feb 2022].

[30] Wasunna M., Musa A., Hailu A., Khalil E.A.G., Olobo J., Juma R. (2016). The Leishmaniasis East Africa Platform (LEAP): strengthening clinical trial capacity in resource-limited countries to deliver new treatments for visceral leishmaniasis. Trans R Soc Trop Med Hyg.

[31] Condon D.L., Beck D., Kenworthy-Heinige T., Bratcher K., O'Leary M., Asghar A. (2017). A cross-cutting approach to enhancing clinical trial site success: The Department of Veterans Affairs' Network of Dedicated Enrollment Sites (NODES) model. Contemp Clin Trials Commun.

[32] Goodacre J., Gibson M., Wilson K., Alfirevic Z., Farrar M., Darbyshire J. (2010). Improved UK clinical trial capacity through the North West Exemplar Programme. Nat Med.

[33] Tupasi T., Gupta R., Danilovits M., Cirule A., Sanchez-Garavito E., Xiao H. (2016). Building clinical trial capacity to develop a new treatment for multidrug-resistant tuberculosis. Bull World Health Organ.

[34] Ogutu B.R., Baiden R., Diallo D., Smith P.G., Binka F.N. (2010). Sustainable development of a GCPcompliant clinical trials platform in Africa: the malaria clinical trials alliance perspective. Malar J..

[35] Salami K., Imbault N., Erlebach A., Urban J., Zoglowek M., Tornieporth N.G. (2020). A systematic scorecard-based approach to site assessment in preparation for Lassa fever vaccine clinical trials in affected countries. Pilot Feasibility Stud.

[36] Berthon-Jones N., Courtney-Vega K., Donaldson A., Haskelberg H., Emery S., Puls R. (2015). Assessing site performance in the Altair study, a multinational clinical trial. Trials.

[37] Gehring M., Taylor R.S., Mellody M., Casteels B., Piazzi A., Gensini G. (2013). Factors influencing clinical trial site selection in Europe: the Survey of Attitudes towards Trial sites in Europe (the SAT-EU Study). BMJ Open.

[38] Seife C. (2015). Research misconduct identified by the US Food and Drug Administration: out of sight, out of mind, out of the peer-reviewed literature. JAMA Intern Med.

[39] Asada R., Yoshimura K., Hattori K., Nonaka Y., Kasai H., Shimizu S. (2019). A descriptive research: exclusion from submitted clinical data package in the review process of new drug approval due to GCP violation in Japan. Contemp Clin Trials Commun.

